# Critical values of learning to speak English for Japanese clinicians

**DOI:** 10.1002/jgf2.487

**Published:** 2021-08-06

**Authors:** Kaku Kuroda, Ryuichi Ohta, R. Eugene Bailey

**Affiliations:** ^1^ Department of Family Medicine SUNY Upstate Medical University Syracuse NY USA; ^2^ Community Care Unnan City Hospital Unnan Japan

## Abstract

We suggest three reasons why Japanese clinicians should learn to speak English: international academic interaction, gaining experience abroad, and having another skill in the rapidly changing world during the COVID‐19 pandemic. Not only reading and writing English but also speaking the language is inevitable for Japanese clinicians. Although speaking English is not easy owing to the disparity between English and Japanese, verbal English fluency adds tremendous value to academic development.

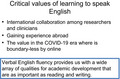

## CONFLICT OF INTEREST

The authors have stated explicitly that there are no conflicts of interest in connection with this article.

To the Editor,

In a recent article, the difficulty that Japanese clinicians have communicating in English in the United States was reviewed.^1^ The author experiences the same struggles in the United States as a family medicine resident.^2^ At the same time, this experience allows me to realize how sophisticated the Japanese language is. Our uniquely complex yet efficient language has played a crucial role in scientific development in Japan, and it has even provided us with our own scientific intellection. Thus, I discussed with my coauthors the question, ‘Should Japanese clinicians learn to speak English?’ This article suggests three insights.

In many foreign medical schools in the countries where English is not the native language, such as Pakistan or Nepal, all medical education is performed in English.^3^ Because this is not the case for Japan, medical education only in Japanese creates a delay of input and output of the newest scientific knowledge worldwide, and less opportunities for international collaboration among researchers and clinicians. In addition, multilingual countries, such as India, need English to serve as a *lingua franca,* which is a language adopted as a common language between speakers whose native languages are different, for their medical communications. Japan is mostly monolingual; thus, most Japanese clinicians do not need to use English as a mutually intelligible language within Japan. However, we cannot discount the fact that international conferences are often conducted in English. For Japanese practitioners to remain relevant on the world stage, we must be able to interact in English and gain our understanding firsthand. In addition, the number of immigrants to Japan is increasing, as well as the opportunity to examine foreign patients for Japanese physicians.^4^

Having an understanding of English can allow clinicians to have international clinical experiences, conduct research, study at school abroad, or present at international conferences. All of these give us a wider academic vision. Regarding research, migrating researchers' articles are cited more often than nonmigrating researchers'.^5^

In this COVID‐19 pandemic, the way doctors should practice medicine has changed drastically. Telemedicine will become a more mainstream practice for many patients. Artificial intelligence will continue to develop to aid the physician and may even replace some medical jobs. More online conferences will be held, shrinking the once‐imposing international distance between countries. In this changing environment, physicians who have another skill—such as speaking English fluently—would be a valuable asset to widen a physician's capability. Knowledge of English is especially valuable, as English is a language spoken widely across the world. If we can use English as a communication tool, we will be able to practice in diverse areas and also broaden clinicians' understanding of social determinants of health, a valuable asset for effective care. Knowledge of English can allow those experiences to come to the physician firsthand instead of through translation.

We conclude that learning to speak English gives us tremendous value for academic development. While reading and writing English is an inevitable and necessary skill for input and output of the newest medical evidence, verbal English fluency provides us with a wider array of qualities that are as important as reading and writing to our practice.
